# Immunological Landscape of Non-Melanoma Skin Neoplasms: Role of CTLA4+IFN-γ+ Lymphocytes in Tumor Microenvironment Suppression

**DOI:** 10.3390/medicina61020330

**Published:** 2025-02-13

**Authors:** Silvana Karabatić Knezović, Dora Knezović, Jelena Ban, Antonela Matana, Neira Puizina Ivić, Merica Glavina Durdov, Mladen Merćep, Irena Drmić Hofman

**Affiliations:** 1 Secondary Medical School Split, 21000 Split, Croatia; silvana.karabatic-knezovic@skole.hr; 2 Laboratory for Cancer Research, School of Medicine, University of Split, 21000 Split, Croatia; 3 Faculty of Biotechnology and Drug Development, University of Rijeka, 51000 Rijeka, Croatia; jelena.ban@biotech.uniri.hr (J.B.); mladen.mercep@biotech.uniri.hr (M.M.); 4 University Department of Health Studies, University of Split, 21000 Split, Croatia; antmatana@ozs.unist.hr; 5 Department of Dermatology, University Hospital of Split, 21000 Split, Croatia; neira.puizina@kbsplit.hr; 6 Department of Pathology, Forensic Medicine and Cytology, University Hospital of Split, 21000 Split, Croatia; mgdurdov@mefst.hr; 7 Department of Pathology, School of Medicine, University of Split, 21000 Split, Croatia; 8 Department of Medical Chemistry and Biochemistry, School of Medicine, University of Split, 21000 Split, Croatia

**Keywords:** non-melanoma skin neoplasms, keratoacanthoma, squamous cell carcinoma, warts, CTLA-4, interferon-gamma

## Abstract

*Background and Objectives*: This study explores the immunological landscapes of non-melanoma skin neoplasms (NMSNs), specifically keratoacanthoma (KA), squamous cell carcinoma (SCC), and common warts (VV). Although benign, KA shares histological similarities with low-grade SCC. The tumor microenvironment (TME) plays a key role in tumor progression, affecting angiogenesis, inflammation, and immune evasion. Viral infections, particularly human papillomavirus (HPV), are linked to NMSN development, with various HPV types identified in KA. VV, caused by HPV, serves as a comparative model due to its similar etiopathogenesis. *Materials and Methods*: This research examines the expression of CTLA4, a critical regulator of T-cell homeostasis, and IFN-γ, a cytokine with immunomodulatory and antiviral effects, in the TME of 41 KA, 37 SCC, and 55 VV samples using multichannel immunofluorescence. *Results*: The analysis revealed distinct patterns of CTLA4 and IFN-γ expression. SCC exhibited a higher prevalence of CTLA4+IFN-γ+ double-positive lymphocytes, suggesting a more immunosuppressive TME. In contrast, VV showed the highest expression of CTLA4+ cells, while both KA and VV had lower expressions of IFN-γ+ lymphocytes compared to SCC. The increased presence of CTLA4+IFN-γ+ double-positive lymphocytes in SCC suggests that the co-expression of these markers may exert a stronger effect on TME modulation than CTLA4 alone. *Conclusions*: These findings underscore the potential of immune profiling as a diagnostic tool to differentiate between benign and malignant lesions, such as KA and SCC. Furthermore, the presence of CTLA4+IFN-γ+ lymphocytes, particularly in SCC, may serve as a biomarker for tumor progression and a potential target for future immunotherapy strategies aimed at modulating the immune response in NMSN.

## 1. Introduction

Non-melanoma skin neoplasms (NMSNs) encompass a variety of keratinocyte-derived tumors that are influenced by factors such as chronic ultraviolet (UV) radiation exposure, age, skin type, and environmental conditions [1,2].

These tumors are increasingly prevalent, with squamous cell carcinoma (SCC) being the second most common type among them [3]. SCC is characterized by histological features such as keratinization, invasion, and nuclear atypia, and is associated with a complex immunological landscape, including tumor-infiltrating lymphocytes and immunosuppressive factors [4].

Unlike SCC, which does not regress spontaneously [5], keratoacanthoma (KA) is a benign lesion that shares histological similarities with low-grade SCC but typically undergoes spontaneous regression. However, approximately 25% of KA cases may progress to SCC [6]. KA develops in three stages: rapid proliferation forming a crater-like nodule, maturation, and eventual regression, which involves apoptosis, though the exact mechanisms remain unclear [7,8]. Major risk factors for KA include UV radiation, human papillomavirus (HPV) infection, trauma, genetic predispositions, and mutations in pathways such as hedgehog signaling and BRAF [9,10].

HPV infections, particularly with beta-HPV types, have been implicated in the development of NMSNs, with various HPV types identified in both KA and common warts (VV) [11,12,13,14,15,16,17]. VV, a benign lesion caused by HPV, shares some etiopathogenic features with KA and serves as a useful model for studying the viral impact on skin neoplasms due to its benign nature and frequent spontaneous resolution [18,19,20].

Studies have shown that HPV-induced cancers, including head and neck squamous cell carcinoma (HNSCC), generally have better prognoses in younger, non-smoker patients [21,22,23]. Lechien et al. attributed this improved prognosis to stronger immune responses, highlighting the roles of innate immune cells like macrophages and Langerhans cells, alongside adaptive immune responses mediated by T regulatory cells, and CD8+ and CD4+ lymphocytes. These immune mechanisms play a critical role in regulating tumor progression [24]. Consequently, therapeutic approaches such as HPV vaccines and immune checkpoint inhibitors (e.g., anti-CTLA4, PD-L1, and PD-L2 antibodies) are becoming increasingly vital in treating HPV-related cancers [25].

Strickley et al. emphasized the critical role of immunity against commensal papillomaviruses in preventing skin cancer development. They showed that the loss of immunity to these commensal HPVs, rather than the oncogenic potential of HPVs themselves, increases the risk of skin cancer in immunosuppressed individuals. Their findings suggest that enhancing T-cell responses against commensal HPVs could provide a novel immune-based approach to preventing skin cancer, especially in patients with compromised immunity. These studies highlight the complex interplay between HPV infection, immune suppression, and skin cancer risk, particularly in immunocompromised patients [26].

The tumor microenvironment (TME) plays a crucial role in the progression and behavior of NMSN, influencing tumor growth, immune evasion, and response to treatment [27,28]. SCC, in particular, is associated with a suppressive TME, often mediated by CTLA4+ and IFN-γ+ lymphocytes [29]. CTLA4 plays a pivotal role in immune evasion by regulating T-cell activation and tolerance, making it a critical target for immunotherapy. Recent studies have underscored the critical role of CTLA4 in immune evasion in a variety of cancer types, including skin cancers. CTLA4 is transiently expressed on activated T cells and permanently expressed on regulatory T cells, and its interaction with its ligands leads to immune suppression by preventing the activation of effector T cells. In recent years, targeting CTLA4 with immune checkpoint inhibitors has shown promising results in enhancing anti-tumor immunity, especially in cancers with a strong immune evasion component, like SCC [25,30,31,32]. Furthermore, interferon-gamma (IFN-γ), a type II interferon with antiviral, antitumor, and immunomodulatory properties, is produced by immune cells such as CD4+ and CD8+ tumor-specific T lymphocytes. IFN-γ is essential for directly suppressing tumor growth. However, IFN-γ also exhibits paradoxical tumor-promoting effects by upregulating the expression of immune checkpoint ligands on tumor cells and T lymphocytes via the JAK-STAT-IRF-1 pathway, thereby complicating immune responses within the TME [33,34].

Despite ongoing research, the precise roles of CTLA4 and IFN-γ in the progression and regression of NMSN are not yet fully understood. The similarities between KA and SCC, as well as between VV and KA regarding viral involvement and spontaneous regression, offer a unique framework for studying immune mechanisms in NMSNs.

In this study, we analyzed the TMEs of SCC, KA, and VV, focusing on CTLA4 expression, a key immune checkpoint regulator, and IFN-γ, a cytokine pivotal to antiviral and antitumor immune responses. By comparing these tumor types, we aim to elucidate their immunological distinctions, identify factors underlying benign versus malignant behavior, and explore implications for novel diagnostic and therapeutic strategies.

## 2. Materials and Methods

This study comprised 41 keratoacanthoma (KA) cases, 37 squamous cell carcinoma (SCC) cases, and 55 common warts (VV) cases from paraffin-embedded tissue samples obtained after surgical excision, all pathologically confirmed for diagnosis.

Inclusion criteria: Histopathological confirmation of KA, SCC, or VV diagnosis, and the availability of high-quality paraffin-embedded tissue samples suitable for immunohistological examination.

Exclusion criteria: Cases with uncertain diagnosis, significant histological overlap between KA, SCC, and other conditions, active bacterial or viral infections at the lesion site, or known immunocompromised states (e.g., HIV/AIDS, immunosuppressive therapy, transplant recipients).

### 2.1. Immunohistological Protocol and Microscopy

Slides, 4 μm thick, were deparaffinized by two 5 min rinses in xylene and rehydrated with descending ethanol concentrations (100% and 95% ethanol). Epitope retrieval was achieved by boiling in Target Retrieval Solution (Tris/EDTA, pH = 9.0, Dako, Glostrup, Denmark) in the microwave, followed by cooling for 15 min. After draining and wiping, samples were circled with a wax pencil. Endogenous peroxidase activity was blocked with hydrogen peroxide (3%) for 10 min, and slides were washed twice in Tris-buffered saline (TBS) and treated with a protein block (1X TBS, 0.025% Tween 20, 5% BSA) for an hour. Primary antibodies, anti-CD4 mouse antibody (4B12, Dako, Glostrup, Denmark) at 1:50 dilution, and anti-CD8 mouse antibody (C8/144B, Dako, Glostrup, Denmark) at 1:100 dilution were applied (50 microliters) and incubated overnight at 4 °C. Subsequently, slides were incubated with labeled polymer (K4065, Dako, Glostrup, Denmark) for 30 min, washed thrice in TBS, and visualized with chromogen DAB+ (K4065, Dako, Glostrup, Denmark) for 10–15 min. Counterstaining with hematoxylin, dehydration, application of mounting medium, and addition of coverslips followed. Three characteristic sites per specimen (tumor center, tumor base, and healthy lateral resection margin) were imaged at 400× magnification using an Olympus BX43 microscope (Olympus, Tokyo, Japan).

### 2.2. Multichannel Immunofluorescence (IF) Protocol and Microscopy

Four-micrometer-thick slides underwent deparaffinization by three 5 min xylene rinses, followed by rehydration with a descending ethanol series (100%, 95%, 70%). Antigen detection involved boiling in citrate buffer (10 mM sodium citrate (Sigma-Aldrich, Burlington, MA, USA), 0.05% Tween 20 (Roth, Karlsruhe, Germany), pH = 6.0) for 12 min in a microwave, and then cooling for 20 min and washing twice with phosphate-buffered saline (PBS) for 5 min. Specimens were circled, wiped, and blocked with a protein block (1X TBS, 0.025% Tween 20, 5% BSA) for 20 min. Primary antibodies (Table 1), at 1:100 dilution, were applied and incubated overnight at 4 °C. After washing twice with PBS for 5 min, secondary antibodies (Table 1) were applied, followed by one-hour incubation and two PBS washes. Cell nuclei were stained with DAPI (1:1000) (Table 1) for double-staining and Hoechst 33,342 (1:500) (Table 1) for triple-staining IF, applied for one and five minutes, respectively, and washed twice with PBS for 5 min. The slides were dried, mounted, cover-slipped, and stored at 4 °C in the dark. For double-staining IF Eukitt (Sigma-Aldrich, Burlington, MA, USA) and for triple-staining the IF Vectashield plus (H-1900, Vector laboratories, Newark, CA, USA) were used as mounting media. An Olympus BX41 fluorescence microscope (Olympus, Tokyo, Japan), Hamamatsu ORCA-Spark camera C11440-36U (Hamamatsu Photonics, Hamamatsu, Japan), Olympus UPLFLN 40× 0.75 numerical aperture objective (Olympus, Tokyo, Japan), and Olympus CellSens Dimension software (version 1.14, Olympus, Tokyo, Japan) were utilized for capturing double-staining fluorescent images. Three characteristic sites per sample (tumor center, tumor base, healthy lateral resection margin) were imaged and analyzed at 400× magnification. Inflammatory cell counts positive for CTLA4 and/or IFN-γ were expressed as a percentage of total inflammatory cells in each field of view and quantified using ImageJ software (version 1.54, NIH, Bethesda, MD, USA). To capture triple-staining fluorescent images, an Olympus IX83 inverted fluorescent microscope (Olympus, Tokyo, Japan) equipped with fluorescence optics (mirror units: U-FUNA: EX360-370, DM410, EM420-460, U-FBW: EX460-495, DM505, EM510IF, and U-FGW: EX530-550, DM570, EM575IF (Olympus) and Cy5 (EX620/60, DM660, EM700/75, Chroma, Bellows Falls, VT, USA)), Hamamatsu Orca R2 CCD camera (Hamamatsu Photonics, Hamamatsu, Japan), and CellSens Dimension software (version 1.14, Olympus, Tokyo, Japan) were used. Three characteristic sites per sample (tumor center, tumor base, healthy lateral resection margin) were imaged using UPLFLN 20× 0.5 numerical aperture objective (Olympus, Tokyo, Japan). All samples were analyzed at 200× magnification using ImageJ (version 1.54, NIH, Bethesda, MD, USA) and CellSens Dimension (version 1.14, Olympus, Tokyo, Japan) software for image processing.

### 2.3. Statistical Analysis

Statistical analysis utilized SPSS Version 28 (SPSS Inc., Chicago, IL, USA). The Shapiro–Wilk test determined the data distribution, and the data were presented via descriptive statistics (median and interquartile range). Chi-square and Fisher’s exact tests were used to compare epidemiological differences, including gender and age. A chi-squared test with assigned probabilities were used to analyze KA localization frequencies. Fisher’s exact test was used to compare age groups and genders regarding KA stages and locations. The Kruskal–Wallis test was used to assess differences in CTLA4+, IFN-γ+, and CTLA4+IFN-γ+ double-positive cell expression among KA localizations, stages, marginal parts of KA, VV, and SCC groups regarding sex and age, and among KA, SCC, and VV considering or pooling data from intratumor characteristic sites. Dunn’s test served as a post hoc analysis. The Mann–Whitney U test was used to compare the expression of the aforementioned cells between sexes for KA, VV, and SCC groups and KA stages. The significance level was set at *p* < 0.05, as it represents a widely accepted standard in biomedical research.

## 3. Results

### 3.1. Epidemiological Characteristics of Cohorts and Pathohistological Characteristics of Keratoacanthoma (KA)

Epidemiological characteristics of patients with keratoacanthoma (KA), squamous cell carcinoma (SCC), and common warts (VV) are shown in Table 2.

This study analyzed patients with KA, SCC, and VV. The age and gender distributions for each group are summarized in Table 2. The groups are evenly distributed concerning the number of lesions (*p* = 0.17) and gender (*p* = 0.338), as determined by chi-square tests. KA and SCC were most prevalent in patients over 79 years, while VV was common in individuals under 48 (*p* < 0.001, Fisher’s exact test). The gender distribution was similar across groups (*p* = 0.338, chi-square test). None of the patients exhibited clinical signs of immunosuppression.

The characteristics of the KA samples (stage and localization) are shown in Table 3.

KA lesions were frequently localized on the head and neck, and extremities, with fewer occurrences on the trunk (*p* = 0.32 and *p* = 0.01, respectively, chi-squared test for given probabilities). The stages of KA included 26 proliferative, 11 regression, and 4 with undetermined stage. No significant differences in CTLA4, IFN-γ, or CTLA4+IFN-γ+ expression were observed across KA localizations (*p* = 0.79, *p* = 0.15, and *p* = 0.37, respectively, Kruskal–Wallis test). Furthermore, there were no differences between age groups and gender regarding KA stages (Fisher’s exact test, *p* = 0.87 and *p* = 0.65, respectively) and localization (Fisher’s exact test, *p* = 0.09 and *p* = 0.34, respectively).

### 3.2. Cross-Sectional CTLA4 and IFN-γ Immunofluorescent Staining of KA, SCC, and VV

**Figure 1:**
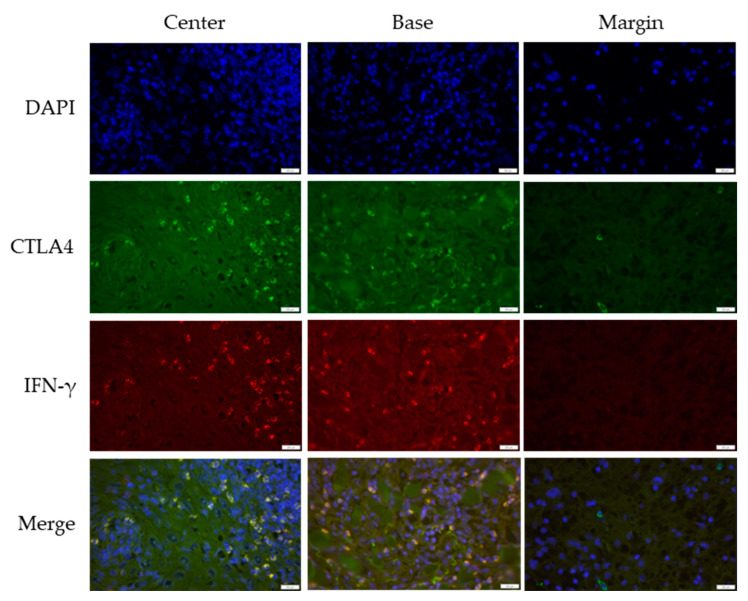
Double immunofluorescent staining of CTLA4 and IFN-γ in keratoacanthoma (KA). The images were acquired using the 40× objective with a 0.75 numerical aperture. DAPI–cell nuclei are stained blue; CTLA4—Cytotoxic T-lymphocyte-associated protein 4, green; IFN-γ—Interferon-gamma, red. Scale bar, 200 µm.

**Figure 2:**
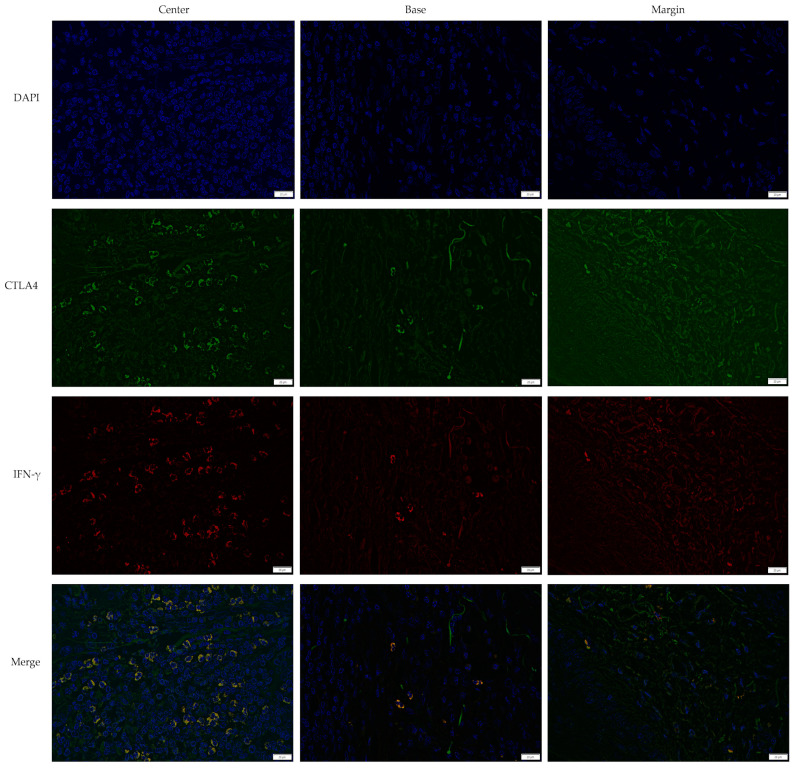
Double immunofluorescent staining of CTLA4 and IFN-γ in squamous cell carcinoma (SCC). The images were acquired using the 40× objective with a 0.75 numerical aperture. DAPI–cell nuclei are stained blue; CTLA4—Cytotoxic T-lymphocyte-associated protein 4, green; IFN-γ—Interferon-gamma, red. Scale bar, 20 µm.

**Figure 3:**
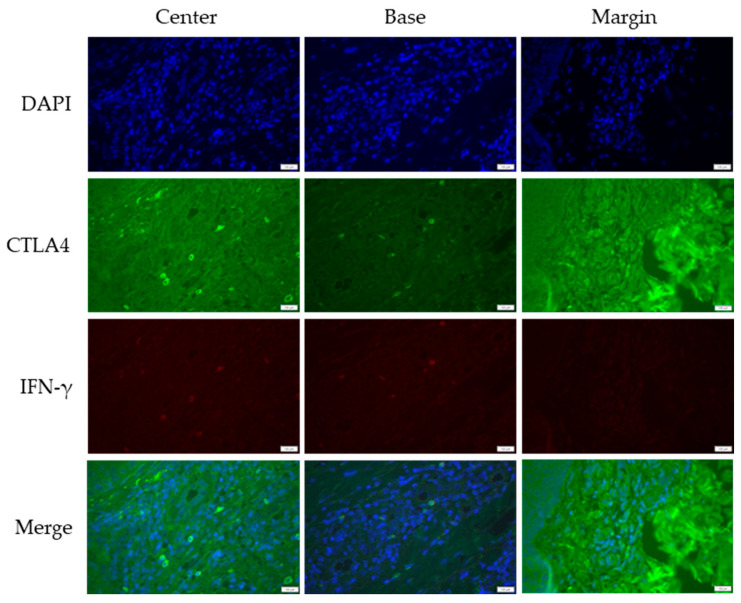
Double immunofluorescent staining of CTLA4 and IFN-γ in common warts (VV). The images were acquired using the 40× objective with a 0.75 numerical aperture. DAPI-cell nuclei are stained blue; CTLA4—Cytotoxic T-lymphocyte-associated protein 4, green; IFN-γ—Interferon-gamma, red. Scale bar, 100 µm.

CTLA4 and IFN-γ expression were analyzed across KA, SCC, and VV using immunofluorescence staining. Figure 1, Figure 2 and Figure 3 illustrate representative staining patterns in characteristic tumor sites (center, base, and margin). The Supplementary Materials (Figures S1–S3) present histological staining and immunohistochemical CD4 and CD8 staining results for KA, SCC, and VV, respectively.

Marginal parts of a lesion were used as controls: In KA and VV, no significant differences in CTLA4 expression were noted based on age and gender, and IFN-γ was not detected. In SCC, patients over 79 exhibited higher IFN-γ+ cell prevalence (*p* = 0.043, Kruskal–Wallis test).

Age-specific differences: There were no differences in the expression of CTLA4+, IFN-γ+, and CTLA4+IFN-γ+ double-positive cells in KA and VV regarding age, while in SCC, patients older than 79 years had a higher prevalence of IFN-γ+ cells (*p* = 0.043), as determined by Kruskal–Wallis’s test.

Gender-specific differences: Women expressed more CTLA4+IFN-γ+ cells in KA (*p* = 0.02) and CTLA4+ cells in SCC (*p* = 0.018), as assessed by Mann–Whitney U test.

### 3.3. Analysis of CTLA4 and IFN-γ Expression in Keratoacanthoma

CTLA4 and IFN-γ expression were analyzed across KA stages using immunofluorescence staining. Supplementary Figures S4–S6 illustrate the representative staining patterns at characteristic tumor sites (center, base, and margin) for the proliferative, regressive, and unknown KA stages, respectively.

**Figure 4:**
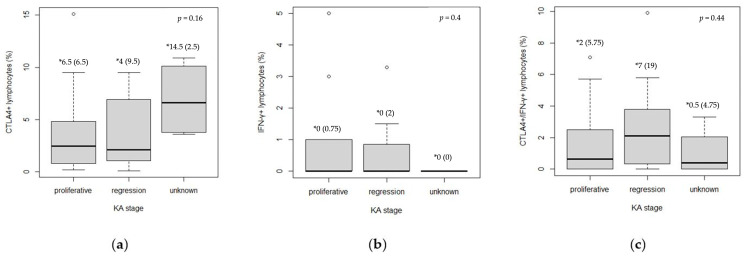
Expression of CTLA4+, IFN-γ+, and CTLA4+IFN-γ+ cells between KA stages (intratumor characteristic sites taken together): (**a**) CTLA4+; (**b**) IFN-γ+; (**c**) CTLA4+IFN-γ+. Kruskal—Wallis test was used to calculate statistical significance. * Median (IQR)—values represent the median and interquartile range of the absolute numbers of positive cells.

Across the KA stages, the regression phase showed significantly higher IFN-γ expression at the lesion base compared to the proliferative phase (*p* = 0.006, Mann–Whitney U test) (Table 4). However, no significant differences were observed when data from all intratumor sites were pooled for CTLA4+, IFN-γ+, and CTLA4+IFN-γ+ cells (Figure 4).

### 3.4. Expression of CTLA4+, IFN-γ+, and CTLA4+IFN-γ+ Cells Between KA, SCC, and VV Tumor Microenvironments

The expressions of CTLA4+, IFN-γ+, and CTLA4+IFN-γ+ cells were compared among KA, SCC, and VV (Table 5).

The key findings include the following:

CTLA4+ expression: VV exhibited significantly higher CTLA4+ cell expression at the center and base compared to KA and SCC (*p* < 0.001, Kruskal–Wallis test, post hoc Dunn test). At the base, KA showed a higher CTLA4+ expression than SCC (*p* < 0.001, Kruskal–Wallis test, post hoc Dunn test). Notably, marginal parts of SCC showed lower CTLA4+ expression compared to KA and VV (*p* < 0.001 and *p* = 0.005, respectively, Kruskal–Wallis test, post hoc Dunn test).

IFN-γ+ expression: Cells positive for IFN-γ only were rare in KA and VV. These lesions showed a lower expression of IFN-γ at the base compared to SCC (*p* = 0.022 and *p* = 0.003, respectively, Kruskal–Wallis test, post hoc Dunn test). Furthermore, IFN-γ+ cells were more prevalent in the SCC margin due to the lack of IFN-γ positivity in marginal parts of KA and VV (*p* < 0.001, Kruskal–Wallis test, post hoc Dunn test).

CTLA4+IFN-γ+ expression: Double-positive cells were notably more prevalent in the central part (*p* < 0.001, Kruskal–Wallis test, post hoc Dunn test), at the base (*p* = 0.002 and *p* < 0.001, respectively, Kruskal–Wallis test, post hoc Dunn test), and in the marginal part (*p* < 0.001, Kruskal–Wallis test, post hoc Dunn test) of SCC compared to KA and VV. KA also exhibited a higher expression of CTLA4+IFN-γ+ cells in the center (*p* = 0.02, Kruskal–Wallis test, post hoc Dunn test) and at the base of lesions (*p* = 0.011, Kruskal–Wallis test, post hoc Dunn test) compared to VV. The results consistently suggest a trend where SCC has the highest expression of CTLA4+IFN-γ+ cells, followed by KA, while VV demonstrates a lower abundance of these cells.

Detailed statistical post hoc Dunn test results are shown in Supplementary Table S1.

**Figure 5:**
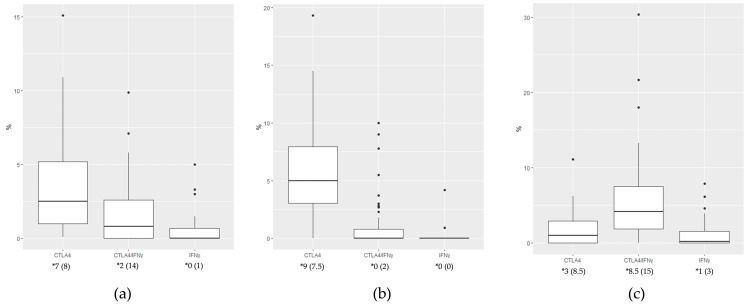
Comparative expression patterns of CTLA4+, CTLA4+IFN-γ+, and IFN-γ+ cells across pooled intratumor sites for (**a**) keratoacanthoma (KA); (**b**) common warts (VV); (**c**) squamous cell carcinoma (SCC). * Median (IQR)—values represent the median and interquartile range of the absolute number of positive cells.

Pooling data from all intratumor sites highlighted that CTLA4+ cells dominated in KA and VV, with IFN-γ+ cells being the least expressed in both groups (*p* < 0.001, Kruskal–Wallis test), while CTLA4+IFN-γ+ double-positive cells were the most abundant type of cells in SCC (*p* < 0.001, Kruskal–Wallis test) (Figure 5).

### 3.5. Defining CTLA4+, IFN-γ+, and CTLA4+IFN-γ+ Cells Using the CD3 Marker for T Lymphocytes in KA, SCC, and, VV Tumor Microenvironments

**Figure 6:**
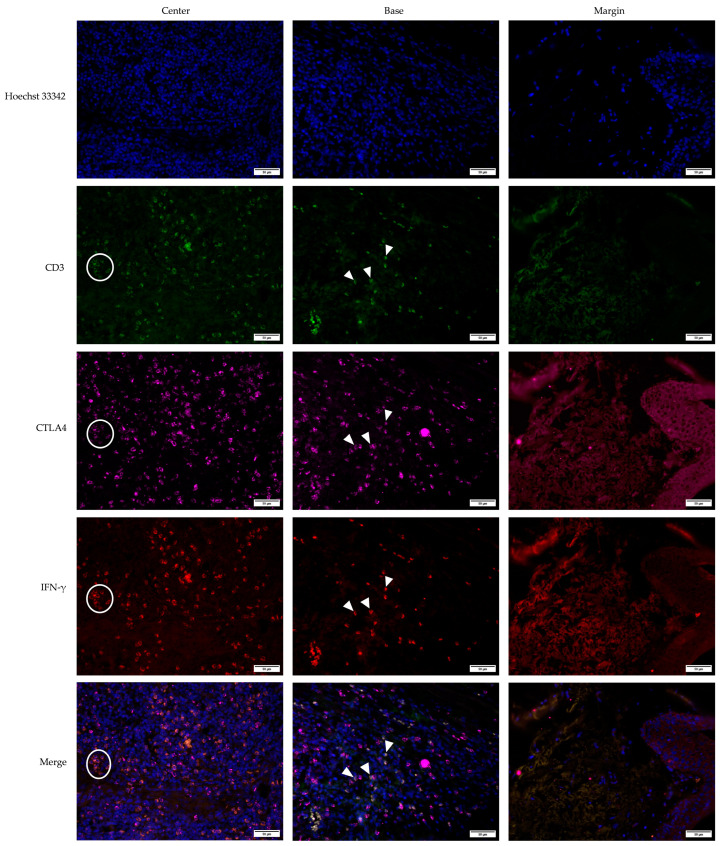
Multichannel immunofluorescent staining of CD3, CTLA4, and IFN-γ in keratoacanthoma (KA). The images are projections of z-stacks acquired with 1.27 μm steps using 20× objective and 0.5 numerical aperture. White arrowheads and circles highlight examples of triple-positive CD3+CTLA4+IFN-γ+ T lymphocytes. Hoechst 33,342 stains cell nuclei blue; CD3—cluster of differentiation 3, green; CTLA4—Cytotoxic T-lymphocyte-associated protein 4, magenta; IFN-γ—Interferon-gamma, red. Scale bar, 50 µm.

**Figure 7:**
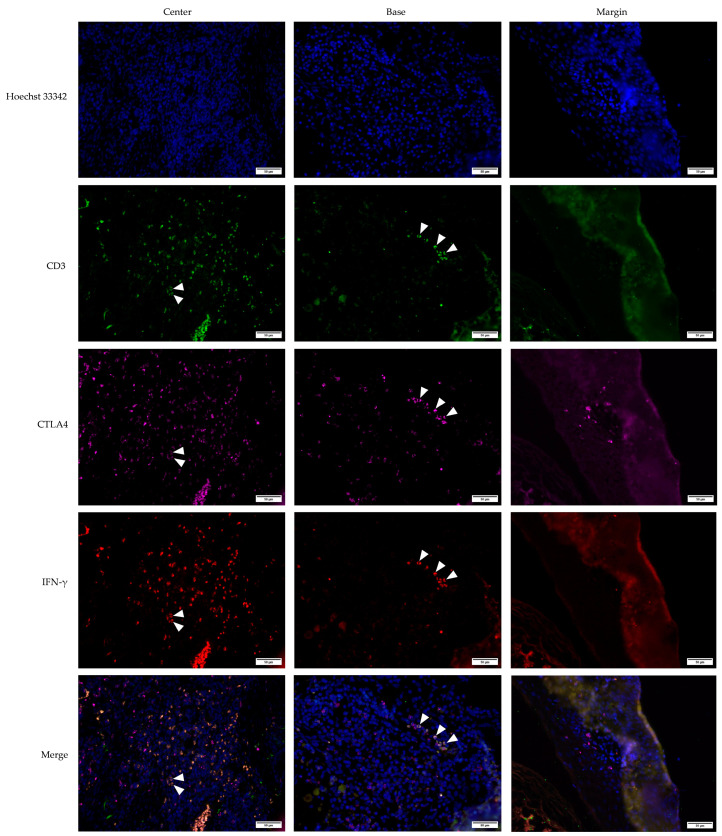
Multichannel immunofluorescent staining of CD3, CTLA4, and IFN-γ in squamous cell carcinoma (SCC). The images are projections of z-stacks acquired with 1.27 μm steps using 20× objective and 0.5 numerical aperture. White arrowheads highlight examples of triple-positive CD3+CTLA4+IFN-γ+ T lymphocytes. Hoechst 33,342 stains cell nuclei blue; CD3—Cluster of differentiation 3, green; CTLA4—Cytotoxic T-lymphocyte-associated protein 4, magenta; IFN-γ—Interferon-gamma, red. Scale bar, 50 µm.

**Figure 8:**
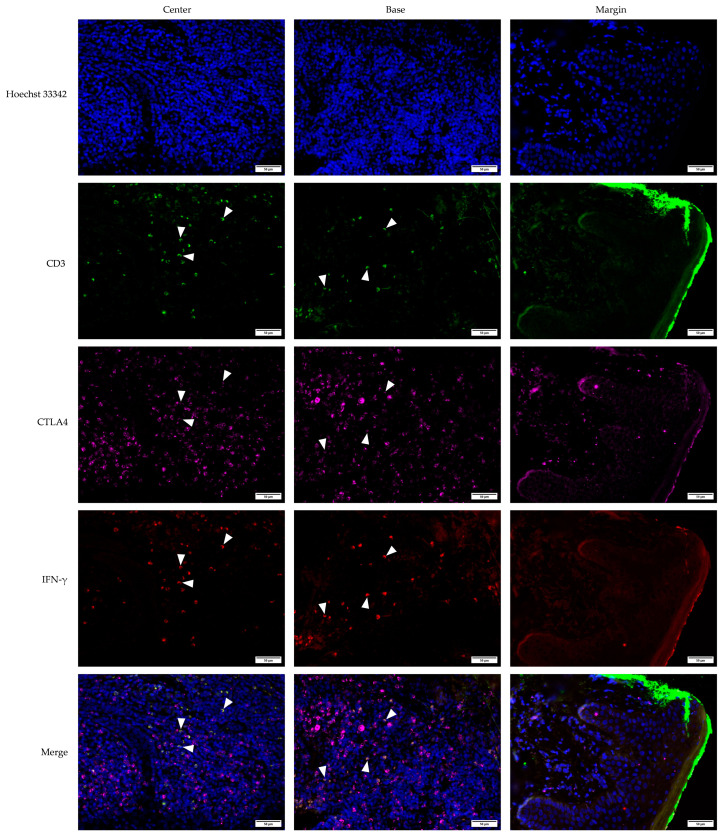
Multichannel immunofluorescent staining of CD3, CTLA4, and IFN-γ in common warts (VV). The images are projections of z-stacks acquired with 1.27 μm steps using 20× objective with a 0.5 numerical aperture. White arrowheads highlight examples of triple-positive CD3+CTLA4+IFN-γ+ T lymphocytes. Hoechst 33,342 stains cell nuclei blue; CD3—Cluster of differentiation 3, green; CTLA4—Cytotoxic T-lymphocyte-associated protein 4, magenta; IFN-γ—Interferon-gamma, red. Scale bar, 50 µm.

Multichannel immunofluorescence staining was used to analyze the KA, SCC, and VV tumor microenvironments. The staining included the CD3 marker to identify T lymphocytes, alongside CTLA4 and IFN-γ markers. This approach allowed for the precise determination of co-expression patterns within the tumor microenvironments of KA (Figure 6), SCC (Figure 7), and VV (Figure 8).

Multichannel immunofluorescence with the T-cell marker CD3 confirmed that the majority of double-positive CTLA4+IFN-γ+ cells across all tumor types were T lymphocytes. All IFN-γ+ cells were CD3+, confirming their identity as T cells (Figure 6, Figure 7 and Figure 8). Interestingly, CTLA4+ cells, particularly in the VV group (Figure 8), frequently lacked CD3 positivity, suggesting a non-T-lymphocyte origin, possibly epithelial cells.

## 4. Discussion

The primary aim of this study was to explore the role of immune modulation in the progression of NMSC, with a particular focus on the immunological interactions mediated by CTLA4+IFN-γ+ double-positive lymphocytes. We examined the tumor microenvironments (TMEs) of keratoacanthoma (KA), squamous cell carcinoma (SCC), and common warts (VV), focusing on two interconnected immune factors: CTLA4, an inhibitory receptor crucial for T-cell homeostasis [32], and IFN-γ, a key antiviral and immunomodulatory cytokine [33]. Our main finding was that SCC progression may be immunologically mediated by suppressive CTLA4+IFN-γ+ double-positive lymphocytes, in contrast to benign lesions such as KA and VV, which typically undergo regression.

Tumor regression, a hallmark of KA, has been the focus of considerable research efforts [35]. Although both KA and VV can regress spontaneously, their recurrence rates differ significantly. While VV demonstrates a high recurrence rate, KA exhibits a local recurrence rate of only 4% [32,36]. Despite differing views, immune cell infiltration into the TME appears critical to both the development and regression of KA [35].

In this study, we explored immune modulation in the TME with a focus on CTLA4 and IFN-γ, which are involved in immune responses in both benign and malignant skin lesions. CTLA4, a crucial immune checkpoint receptor, plays a significant role in T-cell regulation and immune suppression, enabling tumor survival [37,38,39]. Traditionally, CTLA4 has been associated with T-cell dysfunction and the suppression of anti-tumor immunity [40]. Additionally, it is involved in UV-induced immunosuppression, which contributes to the pathogenesis of skin cancers [29]. CTLA4 is not only expressed on T cells, but also on B lymphocytes, natural killer (NK) cells, monocytes, dendritic cells, and even certain tumor cells. This broad expression highlights its importance in various immune interactions within the TME [41].

The role of IFN-γ in tumor biology is complex, exhibiting both anti-tumor and pro-tumor effects. While IFN-γ can directly induce tumor cell apoptosis [42], it may paradoxically promote tumor progression by upregulating PD-L1 expression, which inhibits T-cell cytotoxicity [43]. Furthermore, IFN-γ has been shown to modulate the TME in ways that promote cancer stemness and resistance to therapies [44]. Thus, the dual role of IFN-γ underscores its complex interaction with the tumor immune response [43,44].

Our findings are consistent with previous research on NMSN [5,9,19], with no significant gender differences observed in KA stages. However, more than half of the KA samples were in a proliferative stage, suggesting that a more balanced distribution of KA stages in future studies could offer additional insights. Notably, women exhibited higher levels of CTLA4+IFN-γ+ double-positive lymphocytes in KA and greater CTLA4 expression in SCC. These differences may be attributed to hormonal influences, with estrogen potentially modulating immune suppression in the TME [45] and promoting IFN-γ induction [46]. Age-related differences in immune marker expression were not observed between KA and VV. However, in SCC, patients over 79 displayed higher levels of IFN-γ+ lymphocytes, consistent with previous findings linking aging to increased IFN-γ+ immune response [47,48].

Analysis of immune marker expressions within each group revealed that CTLA4+ cells were more prevalent than CTLA4+IFN-γ+ double-positive and IFN-γ+ cells in both KA and VV, with IFN-γ+ lymphocytes showing the lowest expression. In contrast, SCC showed a predominance of CTLA4+IFN-γ+ double-positive lymphocytes, with no significant difference between CTLA4+ and IFN-γ+ expression. This suggests that while KA and VV share similar immune profiles, SCC displays a distinct immune modulation pattern, particularly with a higher prevalence of immune suppressive markers.

Cross-group comparisons revealed higher CTLA4+ expression in VV compared to both KA and SCC. Since these cells lacked CD3 expression, they are unlikely to be T lymphocytes. It is plausible that these CTLA4+ cells originate from epithelial cells, particularly keratinocytes, which are known to express CTLA4 in response to HPV infection. HPV’s ability to induce CTLA4 expression in keratinocytes is thought to create an immunosuppressive environment that facilitates viral persistence, particularly in the context of common warts. This sustained CTLA4 expression could contribute to the immune evasion mechanisms of HPV, further shaping the TME [49,50,51].

The presence of CTLA4+ cells in benign lesions such as VV suggests that these cells may play a role in modulating T lymphocyte activation and promoting an immunosuppressive environment within the TME. This interplay between CTLA4+ cells and T lymphocytes could be a critical mechanism underlying the immune response in both benign and malignant skin lesions [41,52].

In SCC, IFN-γ+ lymphocytes, identified as CD3+ T cells, were more prevalent across all intratumor sites compared to KA or VV. While IFN-γ typically promotes anti-tumor immune responses, excessive secretion of this cytokine can induce apoptosis in tumor-specific T lymphocytes, facilitating immune escape [53]. Additionally, the abundance of CTLA4+IFN-γ+ double-positive lymphocytes in SCC, which were also CD3+ positive, highlights their role in immune suppression within the TME [54]. Notably, within KA lesions, these double-positive lymphocytes were more prevalent at the lesion center and base compared to VV, indicating a differential immune response across different lesion types.

This study highlights significant differences in CTLA4 and IFN-γ expression between SCC, KA, and VV. The higher prevalence of CTLA4+IFN-γ+ double-positive lymphocytes in SCC suggests a more suppressive TME compared to KA or VV. These findings underscore the potential impact of CTLA4 and IFN-γ co-expression in modulating the TME, which may play a more prominent role in tumor progression than CTLA4 alone.

Despite the promising insights, several limitations should be considered. The relatively small sample size, particularly for specific subtypes such as KA, may limit the generalizability of the findings. While this study focused on key immune markers, it is important to consider other factors and immune cells, including regulatory T cells and tumor-derived exosomes, which may also contribute to the immune response and tumor development in NMSN. Meng et al. emphasize the role of circular RNAs (circRNAs) in regulating immune checkpoints like CTLA4, shaping the TME and modulating resistance to immune checkpoint inhibitors (ICIs) [55].

While ICIs such as those targeting CTLA4 have shown success in melanoma, their application in NMSCs remains an emerging area of research [56]. Future studies should focus on long-term clinical outcomes and mechanisms underlying CTLA4+IFN-γ+ lymphocyte-driven immune suppression, which could potentially improve treatment strategies for NMSN.

Another limitation is the lack of long-term follow-up data, which limits our ability to definitively correlate the observed immune profiles with clinical outcomes, such as recurrence or disease progression. Longitudinal studies tracking patient outcomes over time are crucial for establishing the prognostic significance and therapeutic relevance of CTLA4+IFN-γ+ double-positive lymphocytes.

Our findings suggest a potential link between these double-positive lymphocytes and tumor progression, but further research is required to clarify the exact mechanisms by which these cells contribute to tumor escape and immune resistance. Investigating these pathways could provide valuable insights into the immunopathogenesis of NMSN and reveal new therapeutic targets.

Finally, clinical trials targeting immune checkpoints such as CTLA4 or pathways involving IFN-γ in patients with NMSN could open the door to novel therapeutic interventions. These strategies hold significant promises for enhancing treatment outcomes in skin tumors by modulating the TME and reactivating anti-tumor immune responses.

## 5. Conclusions

This study highlights significant variations in the expression of CTLA4 and IFN-γ across the spectrum of NMSN. The increased prevalence of CTLA4+IFN-γ+ double-positive lymphocytes in SCC suggests a more suppressive TME compared to KA and VV. These findings indicate that the co-expression of CTLA4 and IFN-γ may have a stronger effect on TME modulation than CTLA4 expression alone. Intriguingly, VV expressing more CTLA4+ cells than KA and SCC may reflect a mechanism by which HPV sustains long-term infection, thereby promoting immune evasion and fostering a suppressive TME. The findings suggest that immune profiling could be a valuable tool for distinguishing between benign and malignant lesions, ultimately improving diagnostic accuracy and supporting the development of more targeted therapeutic strategies for NMSNs.

## Figures and Tables

**Table 1:** Primary and secondary antibodies used for multichannel immunofluorescence.

IF Channels		Primary Antibody	Secondary Antibody	Probe
Double-staining	Blue	/	/	DAPI (Sigma-Aldrich, Burlington, MA, USA)
	Green	hCTLA4 rabbit (ab237712, Abcam, Cambridge, UK)	Alexa Fluor 488 anti-rabbit (ab150073, Abcam, Cambridge, UK), 1:400	/
	Red	hIFN gamma mouse IgG2b (ab218426, Abcam, Cambridge, UK)	Rhodamine Red-X AffiniPure Donkey anti-mouse (715-295-151, Jackson ImmunoResearch, West Grove, PA, USA), 1:100	/
Triple- staining	Blue	/	/	Hoechst 33,342 (Fluka, Honeywell, Morris Plains, NJ, USA)
	Green	hCD3 mouse IgG1 (clone F.7.2.38, Dako, Glostrup, Denmark)	Alexa Fluor 488 anti-mouse IgG1 (A21121, ThermoFisher, Waltham, MA, USA), 1:500	/
	Red	hIFN gamma mouse IgG2b (ab218426, Abcam, Cambridge, UK)	CoraLite Plus 555 anti-mouse IgG2b (smsG2bCL555-1, Proteintech, San Diego, CA, USA), 1:500	/
	Far red	hCTLA4 rabbit (ab237712, Abcam, Cambridge, UK)	Alexa Fluor 647 anti-rabbit (ab150083, Abcam, Cambridge, UK), 1:500	/

**Table 2:** Characteristics of patients with keratoacanthoma (KA), squamous cell carcinoma (SCC), and common warts (VV).

Characteristics		KA (n = 41)	SCC (n = 37)	VV (n = 55)
Age (n)	<48	5	0	19
	48–66	10	4	15
	67–79	11	11	12
	>79	15	22	9
Sex (n)	Female	20	12	22
	Male	21	25	33

**Table 3:** Pathohistological and clinical characteristics of keratoacanthoma (KA).

Characteristics		KA (n = 41)
Stage (n)	proliferative	26
	regression	11
	unknown	4
Localization (n)	head and neck	21
	extremities	15
	trunk	5

**Table 4:** Expression of CTLA4+, IFN-γ+, and CTLA4+ IFN-γ+ double-positive cells among KA stages considering intratumor characteristic sites.

		Proliferative	Regression	*p* *
CTLA4+ ^†^ (median (IQR ^‡^))	Center (%)	0.5 (0.7)	0.4 (1.2)	0.70
	Base (%)	1.1 (2.5)	1.2 (1.9)	0.78
	Margin (%)	0.2 (1.2)	0.3 (2.9)	0.47
IFN-γ+ ^§^ (median (IQR))	Center (%)	0.0 (0.1)	0.0 (0.0)	0.64
	Base (%)	0.0 (0.0)	0.0 (0.5)	0.006
	Margin (%)	/	/	/
CTLA4+IFN-γ+ (median (IQR))	Center (%)	0.0 (1.2)	2.1 (2.6)	0.09
	Base (%)	0.3 (0.1)	0.0 (1.7)	0.76
	Margin (%)	/	/	/

* Mann–Whitney U test; ^†^ Cytotoxic T-lymphocyte-associated protein 4; ^§^ Interferon-gamma; ^‡^ Interquartile range.

**Table 5:** Expression of CTLA4+, IFN-γ+, and CTLA4+IFN-γ+ cells across KA, SCC, and VV considering intratumor characteristic sites.

		KA	SCC	VV	*p* *
CTLA4+ median (IQR)	Center (%)	0.6 (0.9)	0.35 (1.03)	1.9 (2.3)	<0.001
	Base (%)	1.2 (2.5)	0.0 (0.55)	2.4 (2.4)	<0.001
	Margin (%)	0.3 (2.1)	0.0 (0.0)	0.0 (1.7)	0.004
IFN-γ+ median (IQR)	Center (%)	0.0 (0.0)	0.0 (0.0)	0.0 (0.0)	0.005
	Base (%)	0.0 (0.0)	0.0 (0.0)	0.0 (0.0)	0.02
	Margin (%)	/	0.0 (1.25)	/	<0.001
CTLA4+IFN-γ+ median (IQR)	Center (%)	0.1 (2.15)	1.44 (2.39)	0.0 (0.7)	<0.001
	Base (%)	0.0 (0.85)	1.45 (2.41)	0.0 (0.0)	<0.001
	Margin (%)	/	0.0 (0.24)	/	<0.001

* Kruskal—Wallis test. Detailed statistical post hoc Dunn test results are shown in Supplementary Table S1.

## Data Availability

The raw data supporting the conclusions of this article will be made available by the authors upon request.

## Supplementary Materials

The following supporting information can be downloaded at https://www.mdpi.com/article/10.3390/medicina61020330/s1, Figure S1: Histological staining and immunohistochemical CD4 and CD8 staining of keratoacanthoma (KA). High-power magnification field (400×). H&E—hematoxylin and eosin, CD4—cluster of differentiation 4, CD8—cluster of differentiation 8. Scale bar, 100 µm; Figure S2: Histological staining and immunohistochemical CD4 and CD8 staining of squamous cell carcinoma (SCC). High-power magnification field (400×). H&E—hematoxylin and eosin, CD4—cluster of differentiation 4, CD8—cluster of differentiation 8. Scale bar, 50 µm; Figure S3: Histological staining and immunohistochemical CD4 and CD8 staining of common warts (VV). High-power magnification field (400×). H&E—hematoxylin and eosin, CD4—cluster of differentiation 4, CD8—cluster of differentiation 8. Scale bar, 100 µm; Figure S4: Double immunofluorescent staining of CTLA4 and IFN-γ in proliferative stage of keratoacanthoma (KA). The images are acquired using the 40x objective with a 0.75 numerical aperture. DAPI—cell nuclei are stained blue; CTLA4—Cytotoxic T-lymphocyte-associated protein 4, green; IFN-γ—Interferon-gamma, red. Scale bar, 200 µm; Figure S5: Double immunofluorescent staining of CTLA4 and IFN-γ in regressive stage of keratoacanthoma (KA). The images are acquired using the 40× objective with a 0.75 numerical aperture. DAPI—cell nuclei are stained blue; CTLA4—Cytotoxic T-lymphocyte-associated protein 4, green; IFN-γ—Interferon-gamma, red. Scale bar, 200 µm; Figure S6: Double immunofluorescent staining of CTLA4 and IFN-γ in keratoacanthoma (KA) where the stage could not be determined (unknown stage). The images are acquired using the 40× objective with a 0.75 numerical aperture. DAPI—cell nuclei are stained blue; CTLA4—Cytotoxic T-lymphocyte-associated protein 4, green; IFN-γ—Interferon-gamma, red. Scale bar, 200 µm; Table S1. Comparison of the expression levels of CTLA4+, IFN-γ+, and CTLA4+IFN-γ+ cells between keratoacanthoma (KA), squamous cell carcinoma (SCC), and common warts (VV) across three distinct intratumor regions (center, base, and margin) using the post hoc Dunn test.

## References

[1] Small J., Barton V., Peterson B., Alberg A.J. (2016). Keratinocyte Carcinoma as a Marker of a High Cancer-Risk Phenotype. Adv. Cancer Res..

[2] Brantsch K.D., Meisner C., Schönfisch B., Trilling B., Wehner-Caroli J., Röcken M., Breuninger H. (2008). Analysis of Risk Factors Determining Prognosis of Cutaneous Squamous-Cell Carcinoma: A Prospective Study. Lancet Oncol..

[3] Eisemann N., Waldmann A., Geller A.C., Weinstock M.A., Volkmer B., Greinert R., Breitbart E.W., Katalinic A. (2014). Non-Melanoma Skin Cancer Incidence and Impact of Skin Cancer Screening on Incidence. J. Investig. Dermatol..

[4] Schmitz L., Kanitakis J. (2019). Histological Classification of Cutaneous Squamous Cell Carcinomas with Different Severity. J. Eur. Acad. Dermatol. Venereol..

[5] Que S.K.T., Zwald F.O., Schmults C.D. (2018). Cutaneous Squamous Cell Carcinoma. J. Am. Acad. Dermatol..

[6] Yus E.S., Simón P., Requena L., Ambrojo P., de Eusebio E. (2000). Solitary Keratoacanthoma. Am. J. Dermatopathol..

[7] Ogita A., Ansai S. (2021). What Is a Solitary Keratoacanthoma? A Benign Follicular Neoplasm, Frequently Associated with Squamous Cell Carcinoma. Diagnostics.

[8] Patil S. (2020). Tumor Immunotherapy—A Lot to Learn from Keratoacanthoma. Med. Hypotheses.

[9] Elder D.E., Massi D., Scolyer R.A., Willemze R. (2018). WHO Classification of Skin Tumours.

[10] Kwiek B., Schwartz R.A. (2016). Keratoacanthoma (KA): An Update and Review. J. Am. Acad. Dermatol..

[11] Forslund O., DeAngelis P.M., Beigi M., Schjølberg A.R., Clausen O.P.F. (2003). Identification of Human Papillomavirus in Keratoacanthomas. J. Cutan. Pathol..

[12] Ekström J., Mühr L.S.A., Bzhalava D., Söderlund-Strand A., Hultin E., Nordin P., Stenquist B., Paoli J., Forslund O., Dillner J. (2013). Diversity of Human Papillomaviruses in Skin Lesions. Virology.

[13] Ekström J., Bzhalava D., Svenback D., Forslund O., Dillner J. (2011). High Throughput Sequencing Reveals Diversity of Human Papillomaviruses in Cutaneous Lesions. Int. J. Cancer.

[14] Arroyo Mühr L.S., Hultin E., Bzhalava D., Eklund C., Lagheden C., Ekström J., Johansson H., Forslund O., Dillner J. (2015). Human Papillomavirus Type 197 Is Commonly Present in Skin Tumors. Int. J. Cancer.

[15] Karagas M.R., Waterboer T., Li Z., Nelson H.H., Michael K.M., Bavinck J.N.B., Perry A.E., Spencer S.K., Daling J., Green A.C. (2010). Genus Human Papillomaviruses and Incidence of Basal Cell and Squamous Cell Carcinomas of Skin: Population Based Case-Control Study. BMJ.

[16] Viarisio D., Mueller-Decker K., Kloz U., Aengeneyndt B., Kopp-Schneider A., Gröne H.-J., Gheit T., Flechtenmacher C., Gissmann L., Tommasino M. (2011). E6 and E7 from Beta Hpv38 Cooperate with Ultraviolet Light in the Development of Actinic Keratosis-Like Lesions and Squamous Cell Carcinoma in Mice. PLoS Pathog..

[17] Viarisio D., Müller-Decker K., Accardi R., Robitaille A., Dürst M., Beer K., Jansen L., Flechtenmacher C., Bozza M., Harbottle R. (2018). Beta HPV38 Oncoproteins Act with a Hit-and-Run Mechanism in Ultraviolet Radiation-Induced Skin Carcinogenesis in Mice. PLoS Pathog..

[18] Plasencia J.M. (2000). Cutaneous warts: Diagnosis and treatment. Prim. Care Clin. Off. Pract..

[19] Bacelieri R., Johnson S.M. (2005). Cutaneous Warts: An Evidence-Based Approach to Therapy. Am. Fam. Physician.

[20] Gogia P., Thami G.P., Poonia K., Bhalla M., Garg G. (2021). Efficacy of Tuberculin Immunotherapy in Verruca Vulgaris: Our Experience from Single Center from North-West India and Review of Literature. Dermatol. Ther..

[21] Ang K.K., Harris J., Wheeler R., Weber R., Rosenthal D.I., Nguyen-Tân P.F., Westra W.H., Chung C.H., Jordan R.C., Lu C. (2010). Human papillomavirus and survival of patients with oropharyngeal cancer. N. Engl. J. Med..

[22] Fakhry C., Westra W.H., Li S., Cmelak A., Ridge J.A., Pinto H., Forastiere A., Gillison M.L. (2008). Improved survival of patients with human papillomavirus-positive head and neck squamous cell carcinoma in a prospective clinical trial. J. Natl. Cancer Ins..

[23] Albers A.E., Qian X., Kaufmann A.M., Coordes A. (2017). Meta analysis: HPV and p16 pattern determine survival in patients with HNSCC and identifies potential new biologic subtype. Sci. Rep..

[24] Lechien J.R., Descamps G., Seminerio I., Furgiuele S., Dequanter D., Mouawad F., Badoual C., Journe F., Saussez S. (2020). HPV Involvement in the Tumor Microenvironment and Immune Treatment in Head and Neck Squamous Cell Carcinomas. Cancers.

[25] Buchbinder E.I., Desai A. (2016). CTLA-4 and PD-1 Pathways. Am. J. Clin. Oncol..

[26] Strickley J.D., Messerschmidt J.L., Awad M.E., Li T., Hasegawa T., Ha D.T., Nabeta H.W., Bevins P.A., Ngo K.H., Asgari M.M. (2019). Immunity to commensal papillomaviruses protects against skin cancer. Nature.

[27] Anderson N.M., Simon M.C. (2020). The Tumor Microenvironment. Curr. Biol..

[28] Chiang E., Stafford H., Buell J., Ramesh U., Amit M., Nagarajan P., Migden M., Yaniv D. (2023). Review of the Tumor Microenvironment in Basal and Squamous Cell Carcinoma. Cancers.

[29] Schwarz A., Beissert S., Grosse-Heitmeyer K., Gunzer M., Bluestone J.A., Grabbe S., Schwarz T. (2000). Evidence for Functional Relevance of CTLA-4 in Ultraviolet-Radiation-Induced Tolerance. J. Immunol..

[30] Zhang C., Chen J., Song Q., Sun X., Xue M., Yang Z., Shang J. (2020). Comprehensive Analysis of CTLA-4 in the Tumor Immune Microenvironment of 33 Cancer Types. Int. Immunopharmacol..

[31] Hosseini A., Gharibi T., Marofi F., Babaloo Z., Baradaran B. (2020). CTLA-4: From Mechanism to Autoimmune Therapy. Int. Immunopharmacol..

[32] Van Coillie S., Wiernicki B., Xu J. (2020). Molecular and Cellular Functions of CTLA-4. Adv. Exp. Med. Biol..

[33] Mojic M., Takeda K., Hayakawa Y. (2017). The Dark Side of IFN-γ: Its Role in Promoting Cancer Immunoevasion. Int. J. Mol. Sci..

[34] Jorgovanovic D., Song M., Wang L., Zhang Y. (2020). Roles of IFN-γ in Tumor Progression and Regression: A Review. Biomark. Res..

[35] Bayer-Garner I.B., Ivan D., Schwartz M.R., Tschen J.A. (2004). The Immunopathology of Regression in Benign Lichenoid Keratosis, Keratoacanthoma and Halo Nevus. Clin. Med. Res..

[36] Savage J.A., Maize J.C. (2014). Keratoacanthoma Clinical Behavior. Am. J. Dermatopathol..

[37] Walunas T.L., Lenschow D.J., Bakker C.Y., Linsley P.S., Freeman G.J., Green J.M., Thompson C.B., Bluestone J.A. (1994). CTLA-4 Can Function as a Negative Regulator of T Cell Activation. Immunity.

[38] Wei S.C., Duffy C.R., Allison J.P. (2018). Fundamental Mechanisms of Immune Checkpoint Blockade Therapy. Cancer Discov..

[39] Sobhani N., Tardiel-Cyril D.R., Davtyan A., Generali D., Roudi R., Li Y. (2021). CTLA-4 in Regulatory T Cells for Cancer Immunotherapy. Cancers.

[40] Fuertes Marraco S.A., Neubert N.J., Verdeil G., Speiser D.E. (2015). Inhibitory Receptors Beyond T Cell Exhaustion. Front. Immunol..

[41] Oyewole-Said D., Konduri V., Vazquez-Perez J., Weldon S.A., Levitt J.M., Decker W.K. (2020). Beyond T-Cells: Functional Characterization of CTLA-4 Expression in Immune and Non-Immune Cell Types. Front. Immunol..

[42] Boulch M., Cazaux M., Cuffel A., Guerin M.V., Garcia Z., Alonso R., Lemaître F., Beer A., Corre B., Menger L. (2023). Tumor-Intrinsic Sensitivity to the pro-Apoptotic Effects of IFN-γ Is a Major Determinant of CD4+ CAR T-Cell Antitumor Activity. Nat. Cancer.

[43] Moon J.W., Kong S.-K., Kim B.S., Kim H.J., Lim H., Noh K., Kim Y., Choi J.-W., Lee J.-H., Kim Y.-S. (2017). IFNγ Induces PD-L1 Overexpression by JAK2/STAT1/IRF-1 Signaling in EBV-Positive Gastric Carcinoma. Sci. Rep..

[44] Gocher A.M., Workman C.J., Vignali D.A.A. (2022). Interferon-γ: Teammate or Opponent in the Tumour Microenvironment?. Nat. Rev. Immunol..

[45] Rothenberger N., Somasundaram A., Stabile L. (2018). The Role of the Estrogen Pathway in the Tumor Microenvironment. Int. J. Mol. Sci..

[46] Lang T.J. (2004). Estrogen as an Immunomodulator. Clin. Immunol..

[47] Sandmand M., Bruunsgaard H., Kemp K., Andersen-Ranberg K., Pedersen A.N., Skinhøj P. (2002). Is Ageing Associated with a Shift in the Balance between Type 1 and Type 2 Cytokines in Humans?. Clin. Exp. Immunol..

[48] Alberti S., Cevenini E., Ostan R., Capri M., Salvioli S., Bucci L., Ginaldi L., De Martinis M., Franceschi C., Monti D. (2006). Age-Dependent Modifications of Type 1 and Type 2 Cytokines within Virgin and Memory CD4+ T Cells in Humans. Mech. Ageing Dev..

[49] Kirk A., Graham S.V. (2024). The human papillomavirus late life cycle and links to keratinocyte differentiation. J. Med. Virol..

[50] Zhou Q., Chen L., Song Y., Ma L., Xiao P., Chen L., Zhen H., Han R., Chen X., Sun S. (2019). Induction of co-inhibitory molecule CTLA-4 by human papillomavirus E7 protein through downregulation of histone methyltransferase JHDM1B expression. Virology.

[51] Dum D., Henke T.L., Mandelkow T., Yang C., Bady E., Raedler J.B., Simon R., Sauter G., Lennartz M., Büscheck F. (2022). Semi-automated validation and quantification of CTLA-4 in 90 different tumor entities using multiple antibodies and artificial intelligence. Lab. Investig..

[52] Pandiyan P., Hegel J.K.E., Krueger M., Quandt D., Brunner-Weinzierl M.C. (2007). High IFN-γ Production of Individual CD8 T Lymphocytes Is Controlled by CD152 (CTLA-4). J. Immunol..

[53] Pai C.-C.S., Huang J.T., Lu X., Simons D.M., Park C., Chang A., Tamaki W., Liu E., Roybal K.T., Seagal J. (2019). Clonal Deletion of Tumor-Specific T Cells by Interferon-γ Confers Therapeutic Resistance to Combination Immune Checkpoint Blockade. Immunity.

[54] Ivanović T., Božić D., Benzon B., Čapkun V., Vukojević K., Glavina Durdov M. (2023). Histological Type, Cytotoxic T Cells and Macrophages in the Tumor Microenvironment Affect the PD-L1 Status of Gastric Cancer. Biomedicines.

[55] Meng L., Wu H., Wu J., Ding P., He J., Sang M., Liu L. (2024). Mechanisms of immune checkpoint inhibitors: Insights into the regulation of circular RNAS involved in cancer hallmarks. Cell Death Dis..

[56] Cranmer L.D., Hersh E. (2007). The role of the CTLA4 blockade in the treatment of malignant melanoma. Cancer Investig..

