# Supplementary material for: Immunological Landscape of Non-Melanoma Skin Neoplasms: Role of CTLA4+IFN-γ+ Lymphocytes in Tumor Microenvironment Suppression
Source: Medicina (Kaunas). 2025 Feb 13;61(2):330. doi: 10.3390/medicina61020330 (PMC11857809; doi:10.3390/medicina61020330)
Supplement: Supplementary file 1 [file medicina-61-00330-s001.zip › Supplementary Table S1.pdf]

Supplementary Table S1. Comparison of the expression levels of CTLA4+, IFN-γ+, and CTLA4+IFN-γ+ cells between keratoacanthoma (KA), squamous cell carcinoma (SCC), and common warts (VV) across three distinct intratumor regions (center, base, and margin) using the post-hoc Dunn test.

Cell type	Intratumor region	KA[a] vs. SCC[b] (p value*)	SCC vs. VV[c] (p value*)	KA vs. VV (p value*)
**CTLA4+[†]**	Center	0,147	< 0,001	< 0,001
	Base	< 0,001	< 0,001	0,04
	Margin	< 0,001	0,005	0,215
**IFN-γ+[§]**	Center	0,424	0,003	0,004
	Base	0,022	0,003	0,274
	Margin	< 0,001	< 0,001	0,5
**CTLA4+IFN-γ+**	Center	< 0,001	< 0,001	0,02
	Base	0,002	< 0,001	0,011
	Margin	< 0,001	< 0,001	0,5

*Each p-value represents the statistical significance of differences in the expression of a specific cell type between two tumor types within a given intratumor region.

a - keratoacanthoma; b - squamous cell carcinoma; c - common warts

† - cytotoxic T-lymphocyte-associated protein 4; § - interferon-gamma
