# Supplementary figures and images for: Immunological Landscape of Non-Melanoma Skin Neoplasms: Role of CTLA4+IFN-γ+ Lymphocytes in Tumor Microenvironment Suppression
Source: Medicina (Kaunas). 2025 Feb 13;61(2):330. doi: 10.3390/medicina61020330 (PMC11857809; doi:10.3390/medicina61020330)

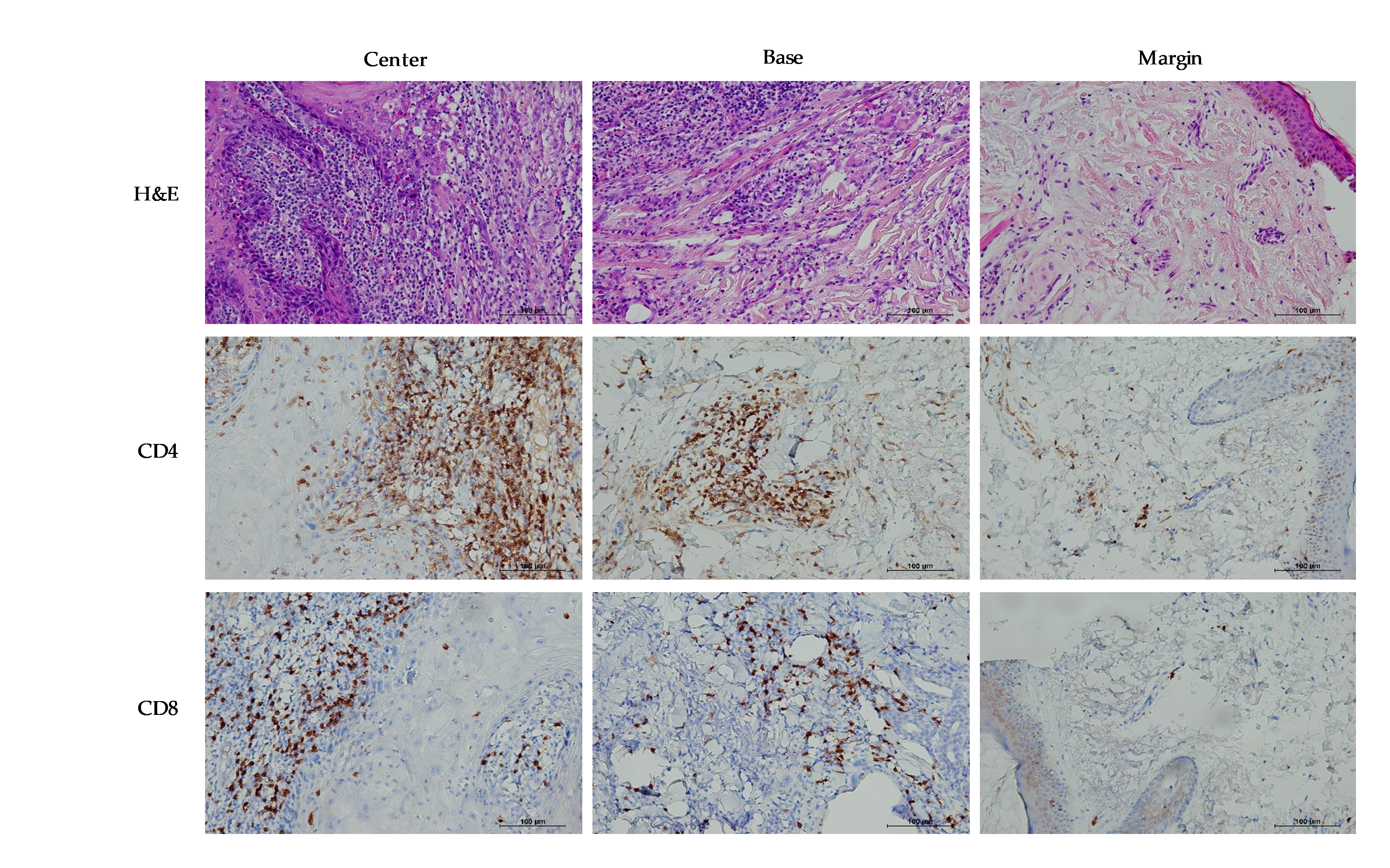

Supplement: Supplementary file 1 [file medicina-61-00330-s001.zip › Figure S1.tif]

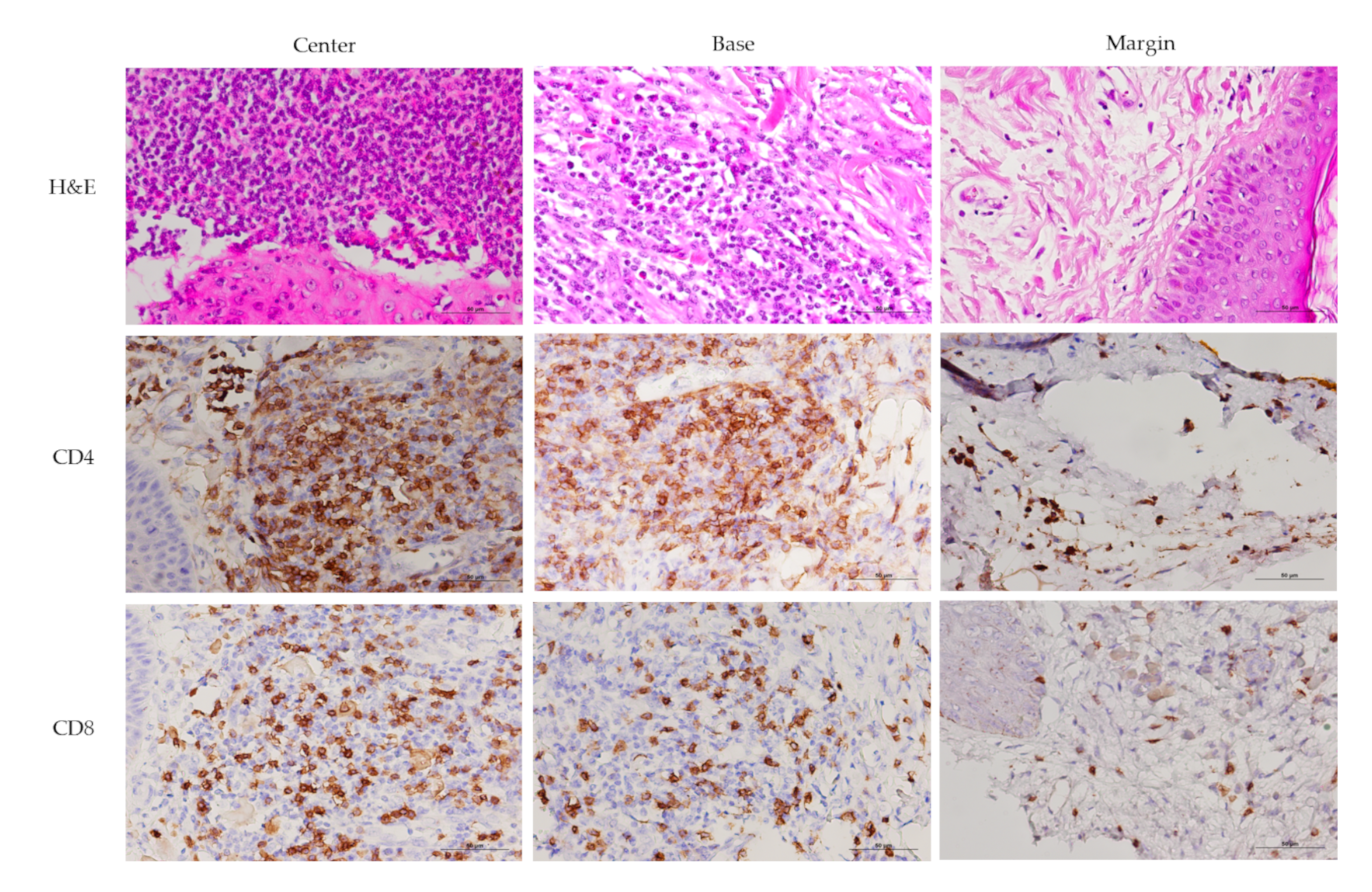

Supplement: Supplementary file 1 [file medicina-61-00330-s001.zip › Figure S2.tif]

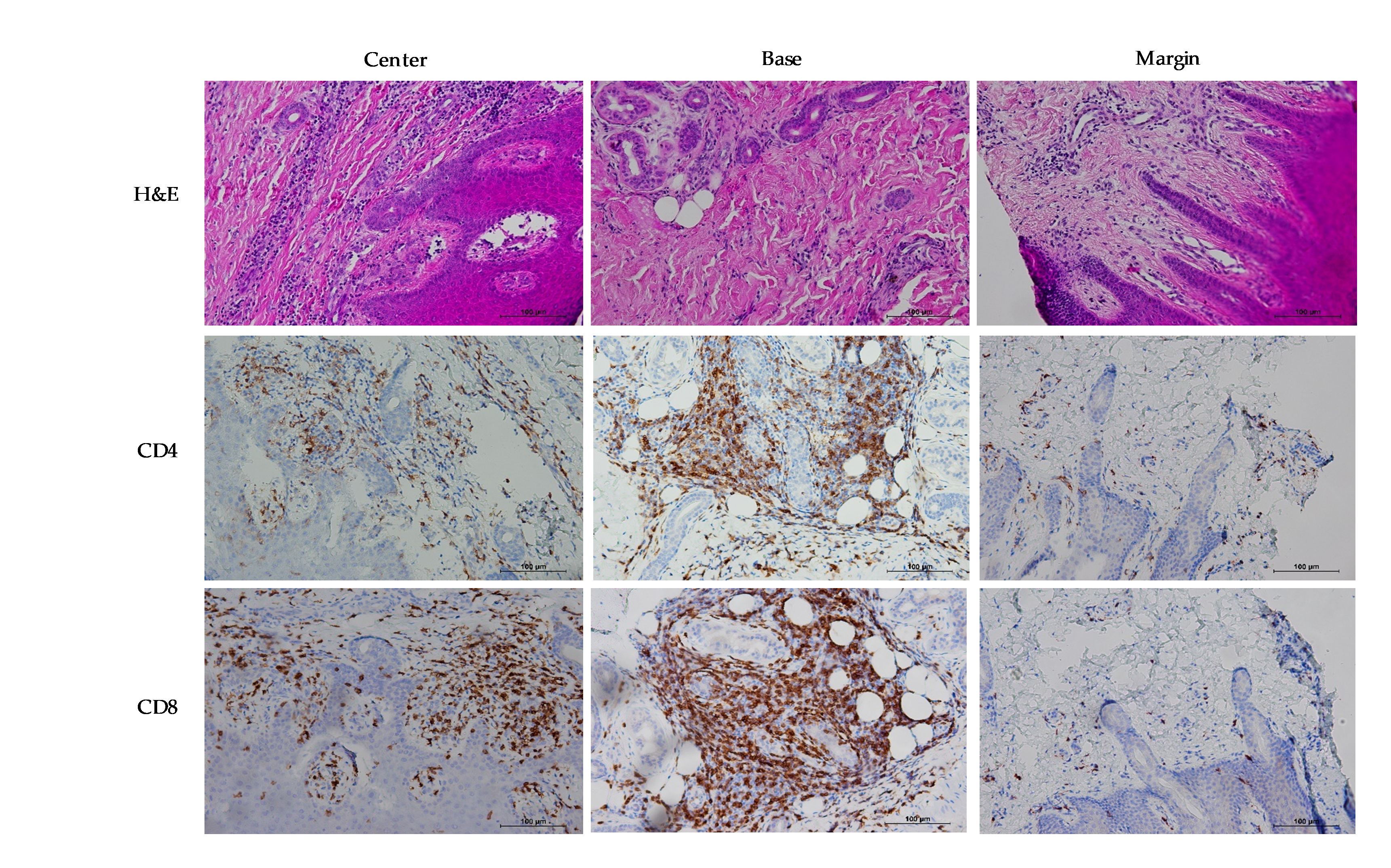

Supplement: Supplementary file 1 [file medicina-61-00330-s001.zip › Figure S3.tif]

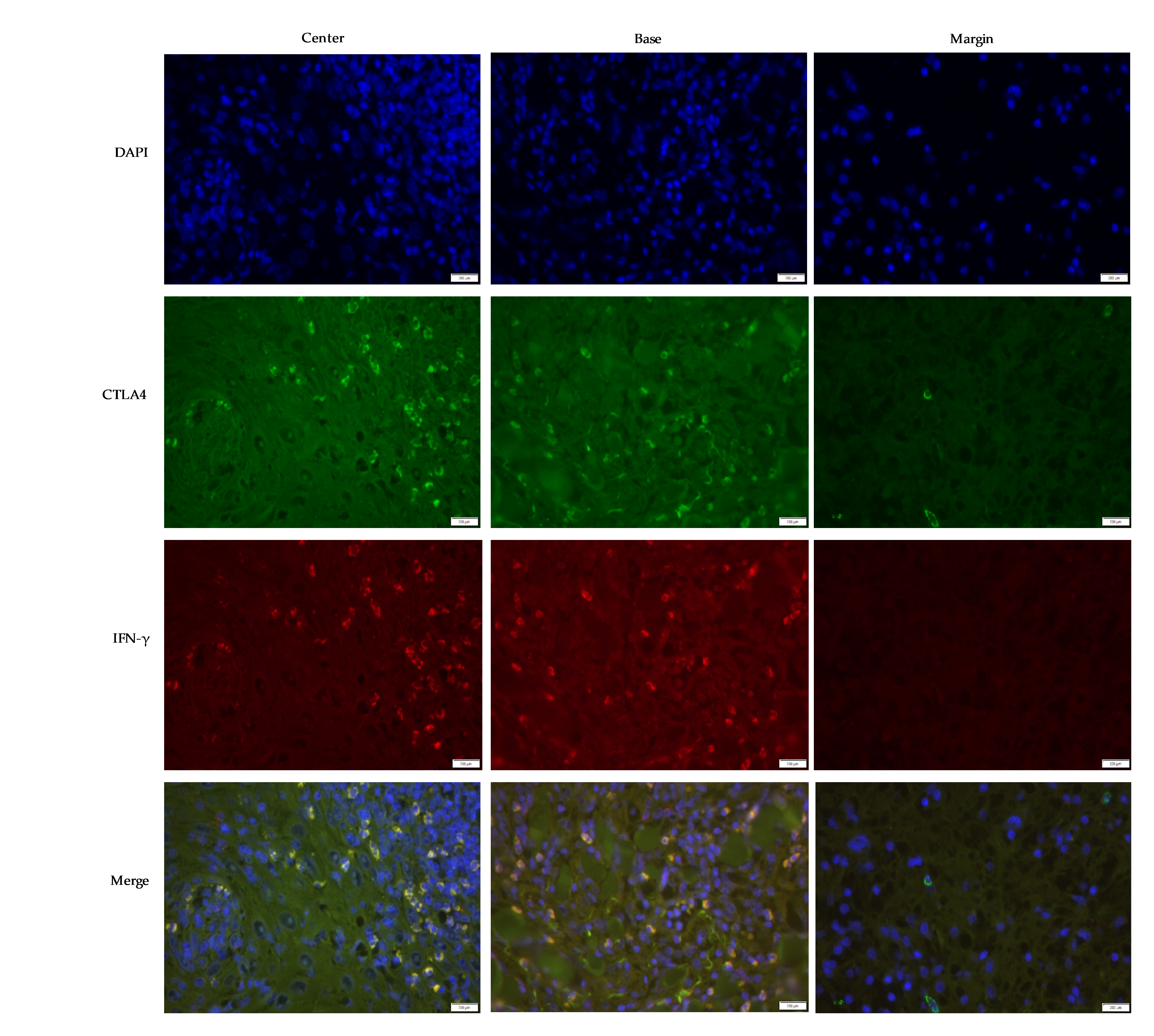

Supplement: Supplementary file 1 [file medicina-61-00330-s001.zip › Figure S5.tif]
